# Cross-Metathesis of Methallyl Halides: Concise Enantioselective Formal Total Synthesis of (–)-Presphaerene

**DOI:** 10.3389/fchem.2020.00494

**Published:** 2020-06-30

**Authors:** Suresh Mandava, Jaun Koo, Jungjoong Hwang, Hari Krishna Nallapaneni, Haeil Park, Jongkook Lee

**Affiliations:** Pharmaceutical Chemistry Laboratory, College of Pharmacy, Kangwon National University, Chuncheon, South Korea

**Keywords:** cross-metathesis, methallyl halide, Stewart–Grubbs catalyst, total synthesis, presphaerene

## Abstract

The cross-metathesis (CM) of methallyl halides catalyzed using four different ruthenium-based complexes—Grubbs catalyst, Grubbs second-generation catalyst, Hoveyda-Grubbs second-generation catalyst, and Stewart–Grubbs catalyst—was investigated. When methallyl chloride or bromide was reacted with a model substrate containing a benzyl ether group, the Grubbs catalyst, and Grubbs second-generation catalyst did not promote the reaction well. However, the Hoveyda–Grubbs second-generation catalyst and Stewart–Grubbs catalyst afforded the corresponding products in moderate to good yield with moderate *E*/*Z* selectivity. Accordingly, several functionalized methallyl halides were prepared by CM. Various functional groups were well-tolerated in this system when the Stewart–Grubbs catalyst was used. To demonstrate the practical utility of our method, methallyl halide CM was successfully employed for the formal total synthesis of a natural product (–)-presphaerene, in which the precursor of the key cyclopentanecarboxylate intermediate was efficiently prepared in three steps.

## Introduction

**Figure 1:**
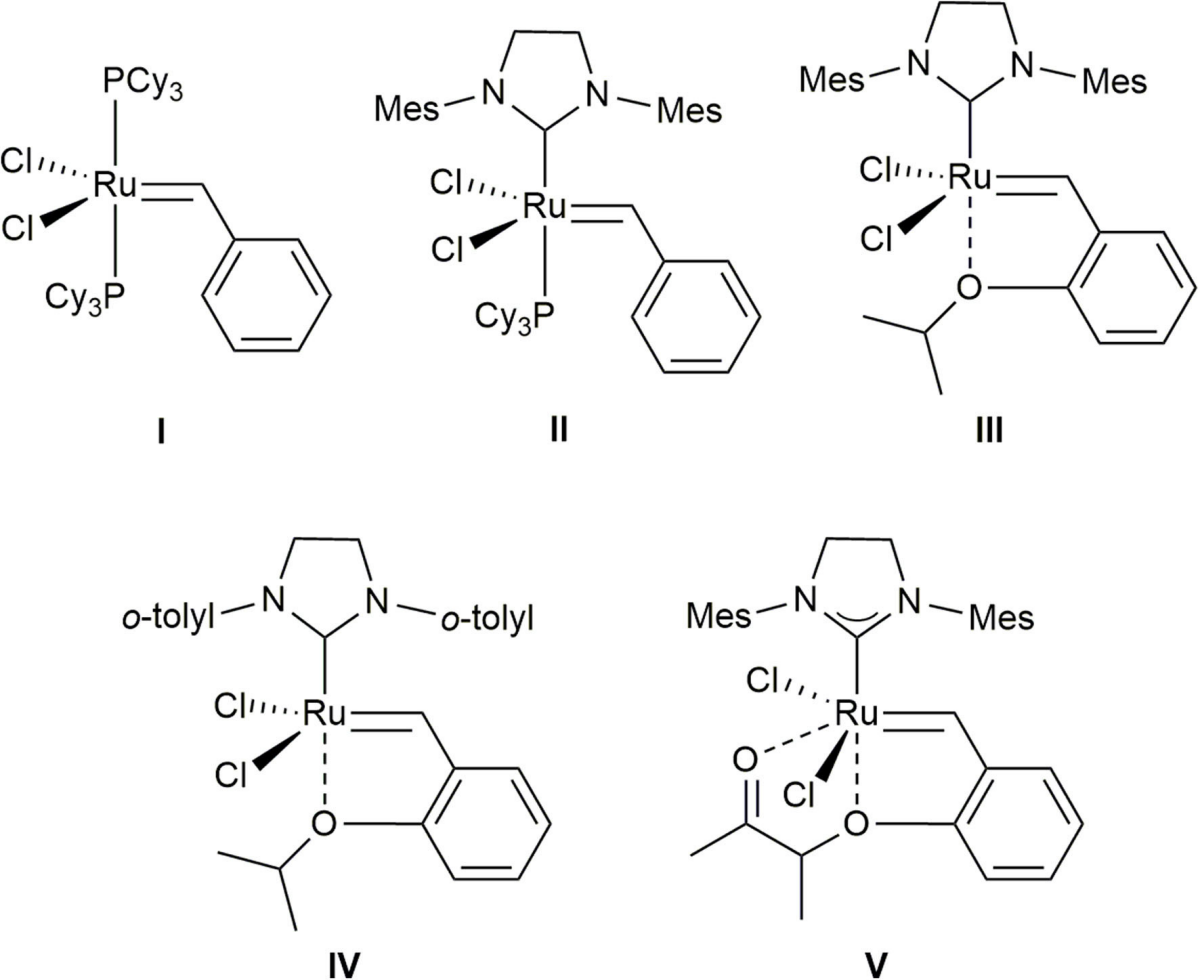
Grubbs catalyst (**I**), Grubbs second-generation catalyst (**II**), Hoveyda–Grubbs second-generation catalyst (**III**), Stewart–Grubbs catalyst (**IV**), and Umicore^TM^ M51 (**V**) considered in this study.

Currently, methallyl halide moieties are typically introduced by conversion of a preexisting aldehyde via olefination–reduction–halogenation (Phoenix et al., 2008; Tanabe et al., 2014; Kauhl et al., 2016) or Grignard addition–halogenation sequences (Davies et al., 2010; Ghosh and Li, 2011), especially in natural product synthesis. Alternatively, the cross-metathesis (CM) of methallyl halides, which would produce trisubstituted alkenes (Nguen et al., 2017; Xu et al., 2017), has the possibility to serve as a useful synthetic tool for the incorporation of methallyl halide moieties into organic molecules. However, despite its potential, this methodology has been hitherto poorly investigated. Accordingly, very few studies on the CM of methallyl halides using ruthenium-based catalysts **I**–**V** (Figure 1) (Schwab et al., 1995; Kingsbury et al., 1999; Scholl et al., 1999; Garber et al., 2000; Stewart et al., 2008) have been published. As rare examples, the Hoveyda–Grubbs second-generation catalyst (**III**) has been used to promote the CM of methallyl chloride with α-methylene-β-lactam to produce the corresponding tetrasubstituted alkene in moderate yield with moderate selectivity (*E*/*Z*, 1:1.6) (Liang et al., 2009). Several functionalized olefins have been shown to smoothly undergo CM with methallyl chloride when ruthenium-based complex **V** (Umicore^TM^ M51) is employed as the catalyst (Bilel et al., 2014).

Although the CM of methallyl halides could serve as a synthetic shortcut, substituting a single step for the numerous functional-group transformations required for the corresponding conventional sequences, this reaction has not been hitherto successfully applied to natural product synthesis. For instance, an attempt to introduce a methallyl chloride moiety into a complex olefin for the total syntheses of the natural products stephacidins A and B and notoamide B using ruthenium-based catalysts **I** and **II** did not afford the corresponding product (Artman III et al., 2007).

**Figure 2:**
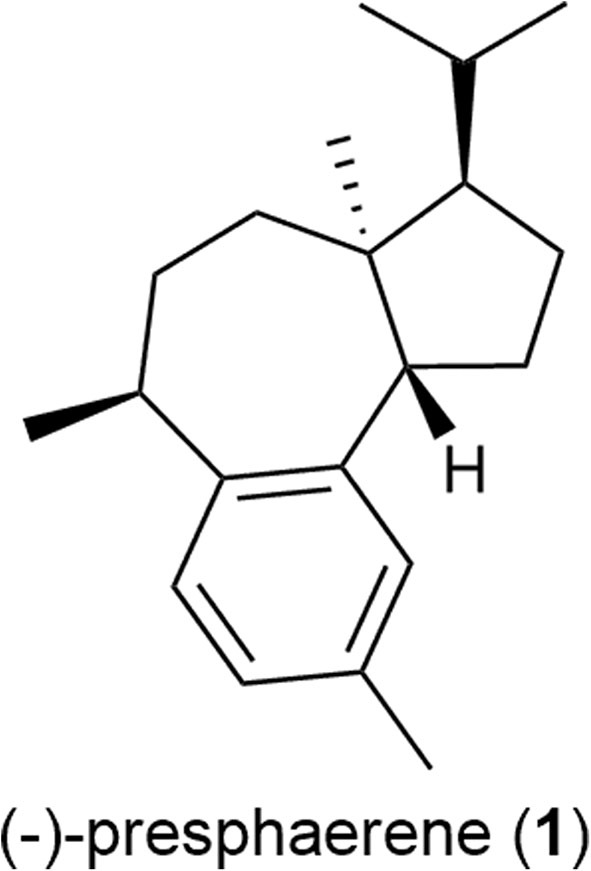
Structure of (–)-presphaerene (**1**).

Accordingly, we herein investigated the ruthenium-complex-catalyzed CM of methallyl halides with several functionalized olefins and its application to the formal total synthesis of a sphaeroane diterpene, (–)-presphaerene (**1**) (Figure 2) (Cafieri et al., 1983).

## Materials and Methods

Experimental procedures and compound characterization data are provided in the Supplementary Material.

## Results and Discussion

We first attempted the CM of methallyl chloride with commercially available olefin **2** bearing a benzyl ether group in CH_2_Cl_2_ employing ruthenium-based catalysts **I–IV**. While the Grubbs catalyst (**I**) and Grubbs second-generation catalyst (**II)** catalyze the reaction poorly, both **III** and Stewart–Grubbs catalyst (**IV**) promote the CM of methallyl chloride with **2** in good yield and with moderate *E*/*Z* selectivity (Table 1, entry 1). Excess amounts of methallyl chloride were required to reduce homocoupling of olefin **2** and to completely convert **2** to the corresponding product.

**Table 1:** Cross-metathesis (CM) of methallyl halides with olefin **2**^a^.

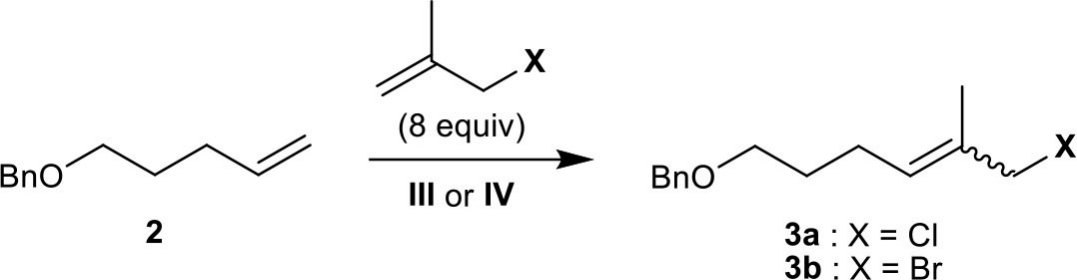					
**Entry**	**X**	**Solvent**	**Product**	**Yield**^**b**^ **[%, (*****E*****/*****Z*****)**^**c**^**]**	
				**III**^**d**^	**IV**^**d**^
1	Cl	CH_2_Cl_2_	**3a**	60 (2.8:1)	64 (3.2:1)
2	Cl	C_2_H_4_Cl_2_	**3a**	35 (2.8:1)	40 (3.6:1)
3	Cl	EtOAc	**3a**	50 (3.0:1)	58 (3.0:1)
4	Cl	THF	**3a**	33 (1.9:1)	55 (3.4:1)
5	Cl	benzene	**3a**	55 (3.4:1)	63 (3.7:1)
6	Cl	toluene	**3a**	55 (3.0:1)	78 (3.3:1)
7	Br	CH_2_Cl_2_	**3b**	19 (1.6:1)	21 (1.3:1)
8	Br	C_2_H_4_Cl_2_	**3b**	19 (1.7:1)	16 (1.4:1)
9	Br	EtOAc	**3b**	32 (1.7:1)	41 (1.9:1)
10	Br	THF	**3b**	23 (1.9:1)	31 (2.0:1)
11	Br	benzene	**3b**	41 (1.8:1)	55 (1.8:1)
12	Br	toluene	**3b**	43 (2.0:1)	68 (2.0:1)

a *All reactions were performed with olefin **2** (0.2 mmol) in solvents (0.1 M) for 18 h at 40°C under an argon atmosphere*.

b *Isolated yield*.

c *The ratio was determined by the analysis of ^1^H 400 MHz NMR spectra*.

d *Total 20 mol% (time 0, 10 mol%; time 5 h, 10 mol%) of **III** or **IV** was used to complete the reaction*.

The reaction was then performed in several other solvents, including EtOAc, THF, benzene, and toluene (Table 1, entries 2–6). The methallyl chloride CM of **2** proceeds over **IV** in benzene and toluene with the highest *E*/*Z* selectivity and yield, respectively.

The CM of methallyl bromide with **2** was also conducted in several solvents including CH_2_Cl_2_, EtOAc, THF, benzene, and toluene. In contrast to the CM of methallyl chloride, the CM of methallyl bromide proceeds poorly under most conditions assessed (Table 1, entries 7–11). Only catalyst **IV** in toluene promotes the methallyl bromide CM of **2** smoothly and in good yield with moderate *E*/*Z* selectivity (Table 1, entry 12). The relatively poor selectivity compared to the corresponding methallyl chloride CM might be attributed to the instability of the *E*-form of **3b**. Nevertheless, to the best of our knowledge, this constitutes the first example of the incorporation of a methallyl bromide moiety into a functionalized olefin by the CM of methallyl bromide.

The CM of methallyl iodide with **2** was also attempted under a variety of conditions in the presence of **III** or **IV**, but the reaction was not observed to any appreciable extent.

Next, we investigated the tolerance of methallyl halide CM to different functional groups, including alkyl, aryl, ester, ketone, hydroxyl, silyl, and epoxy groups, under similar conditions to those employed for the methallyl halide CM of **2**.

The methallyl halide CM of commercially or readily available substrates **4a–h** (Brimble et al., 2005) is well-catalyzed by **IV** (Table 2, Table S1). The reactions exhibit moderate selectivity (*E*/*Z*, 1.7–4.6:1) in most cases, but the CM of methallyl bromide with both ketone **4d** and silyl ether **4f** affords the corresponding functionalized methallyl bromides **15d** and **15f** with poor selectivity (Table S1, entries 4 and 6). The methallyl chloride CM reactions of **4a–h** show higher performances than their corresponding methallyl bromide counterparts with respect to both yield and *E*/*Z* selectivity. This high performance may be attributed to the better stability of functionalized methallyl chlorides **5a–h** than those of the corresponding functionalized methallyl bromides **15a–h** considering almost complete conversion of **4a–h**.

**Table 2:** CM of methallyl chloride with functionalized olefins^a^.

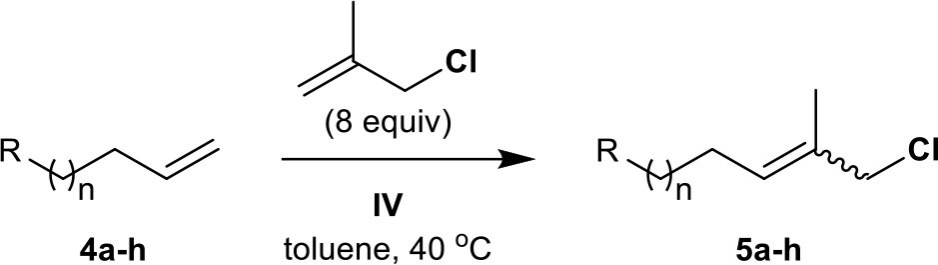						
**Entry**	**Compound**	**R–**	***n***	**Reaction time (*****h*****)**	**Yield (%)**^**b**^	**Ratio (*****E*****/*****Z*****)**^**c**^
1	**4a**	Me–	8	24	**5a**, 80	2.0:1
2	**4b**	AcO–	2	18	**5b**, 81	3.8:1
3	**4c**	EtO_2_C–	1	24	**5c**, 83	4.6:1
4	**4d**	Ac–	1	36	**5d**, 77	3.1:1
5	**4e**	HO–	7	18	**5e**, 75	3.8:1
6	**4f**	TBSO–	7	18	**5f**, 81	2.2:1
7	**4g**	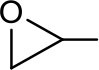	3	18	**5g**, 83	3.4:1^d^
8	**4h**	*p*-MeOPh–	0	24	**5h**, 77	3.8:1

a *Total 20 mol% (time 0, 10 mol%; time 5 h, 10 mol%) of **IV** was used to complete the reaction*.

b *Isolated yield*.

c *The ratio was determined by the analysis of ^1^H 400 MHz NMR spectra*.

d *The ratio was determined by the analysis of ^1^H 600 MHz NMR spectra*.

We next directed our attention to the CM of methallyl halides with commercially available olefins that possess nitrogen-containing functional groups, including amide, imide, carbamate, and nitrile groups. In our previous studies, the *N,N*-dimethylamide group was intolerant to allyl halide CM, but the electron-deficient Weinreb amide group tolerated the reaction catalyzed by **III** (Yun et al., 2011, 2012).

The methallyl halide CM of olefins bearing amide groups was first examined. Both Weinreb amide and *N,N*-dimethylamide groups tolerate methallyl halide CM when **IV** is employed as the catalyst (Table 3, entries 1 and 2), while ruthenium complex **III** serves as a poor catalyst for the methallyl halide CM of *N,N*-dimethylamide **6b** (data not shown). As expected, the methallyl halide CM of Weinreb amide **6a** proceeds more effectively than that of **6b** with respect to yield and selectivity (both Table 3 and Table S2, entries 1 and 2). Catalyst **IV** promotes the CM of methallyl halides with phthalimide **6c** in good yield and with moderate *E*/*Z* selectivity (both Table 3 and Table S2, entry 3). Both carbamate and nitrile groups tolerate methallyl chloride CM (Table 3, entries 4 and 5) but not methallyl bromide CM (Table S2, entries 4 and 5), most likely due to the instability of functionalized methallyl bromides **16d**, **e**. The methallyl chloride CM reactions of **6a–d** are also superior to the corresponding methallyl bromide CM reactions with respect to yield.

**Table 3:** CM of methallyl chloride with olefins bearing nitrogen-containing functional groups^a^.

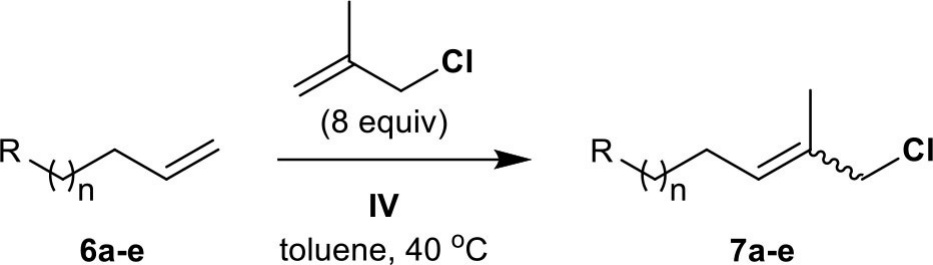						
**Entry**	**Compound**	**R–**	***n***	**Reaction time (*****h*****)**	**Yield (%)**^**b**^	**Ratio (*****E*****/*****Z*****)**^**c**^
1	**6a**	Me(MeO)N(O)C–	3	24	**7a**, 83	4.6:1^d^
2	**6b**	Me_2_N(O)C–	3	36	**7b**, 77	3.1:1
3	**6c**	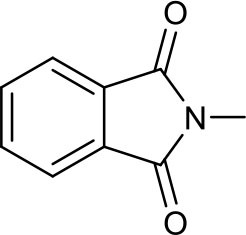	2	18	**7c**, 81	3.8:1
4	**6d**	BocNH–	2	18	**7d**, 63	3.6:1
5	**6e**	NC–	3	24	**7e**, 79	3.4:1^d^

a *Total 20 mol% (time 0, 10 mol%; time 5 h, 10 mol%) of **IV** was used to complete the reaction*.

b *Isolated yield*.

c *The ratio was determined by the analysis of ^1^H 400 MHz NMR spectra*.

d *The ratio was determined by the analysis of ^1^H 600 MHz NMR spectra*.

**Figure 3:**
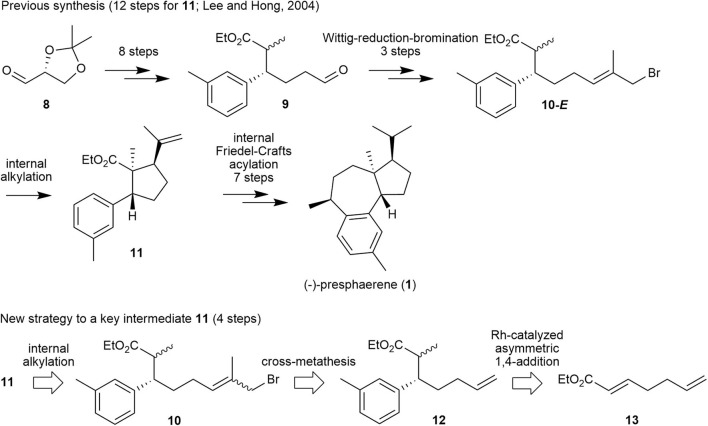
Our previous synthetic approach to (–)-presphaerene (**1**) and a new retrosynthetic approach to its key intermediate **11**.

Encouraged by these results, we prepared a retrosynthetic plan for the formal total synthesis of **1** in combination with our previously published total synthesis of this natural product (Lee and Hong, 2004) as a means to demonstrate the efficiency of methallyl halide CM. Specifically, the first total synthesis of compound **1**, which we published in 2004, proceeds over 19 steps from glyceraldehyde **8** with internal alkylation and intramolecular Friedel–Crafts acylation as key steps, as shown in Figure 3. In this scheme, cyclopentanecarboxylate **11** is cyclized from its precursor **10-*E***, in which the methallyl bromide moiety is introduced via a Wittig reaction–reduction–bromination sequence from aldehyde **9**.

We envisioned that methallyl bromide CM could provide a synthetic shortcut to the internal alkylation substrate by substituting several functional-group transformations in the sequence. Specifically, internal alkylation substrate **10** could be prepared from olefin **12**, which could be generated from α,β-unsaturated ester **13** by utilizing Rh-catalyzed asymmetric 1,4-addition and subsequent methylation, by methallyl bromide CM.

**Figure 4:**
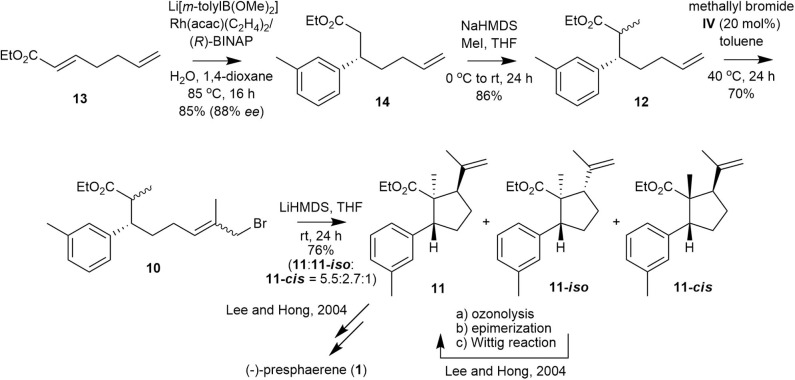
Formal total synthesis of (–)-presphaerene (**1**).

The synthesis commenced with the preparation of a Michael adduct (Figure 4). Commercially available α,β-unsaturated ester **13** undergoes smooth asymmetric 1,4-addition with lithium *m*-tolylborate, which is generated *in situ* from 3-bromotoluene, *n*-butyllithium and trimethoxyborane, in the presence of Rh(acac)(C_2_H_4_)_2_/(*R*)-BINAP to produce ester **14** in 85% yield and 88% *ee* (Takaya et al., 1999; Hayashi, 2001; Hayashi and Yamasaki, 2003). The *ee* of **14** was determined by chiral HPLC analysis (Phenomenex Lux Cellulose-4 column) of the corresponding alcohol reduced from ester **14**. Ester **14** is smoothly methylated to **12** as a diastereomeric mixture (3:2, ^1^H 400 NMR analysis) in 86% yield.

With olefin **12**, we performed methallyl bromide CM to obtain the precursor of cyclopentanecarboxylate **11**. Catalyst **IV** catalyzed the methallyl bromide CM of **12** to afford ω-bromoester **10**, which was subjected to the next step without separation of all the isomers, in 70% yield with moderate *E*/Z selectivity (~3:1, according to ^1^H 600 MHz NMR analysis). ω-Bromoester **10** was treated with LiHMDS in THF for 24 h at room temperature to yield cyclopentanecarboxylate **11**, a key cyclized intermediate in the synthesis of **1**, along with **11-*iso***, which can be converted to **11** as reported in our previous synthesis, and **11-*cis***in a 76% total yield with 5.5:2.7:1 stereoselectivity (Lee and Hong, 2004).

The lower stereoselectivity and yield reported here compared with those of our previous synthesis (5.5:2.7:1 vs. 9.9:3.3:1, 76 vs. 86%) may be attributed to the poorer internal alkylation of the *Z-*isomer of **10** (**10-*Z***) compared with that of the *E*-isomer of **10** (**10-*E***). In our first synthesis of **1**, only the *E*-isomer of **10** (**10-*E***) was used as an internal alkylation substrate.

We obtained the highly functionalized cyclopentanecarboxylate **11**, a key intermediate of **1** that was previously synthesized from glyceraldehyde **8** via 12 steps and in 16% overall yield, from heptadienoate **13** via four steps in 23% overall yield. Thus, **1** may be obtained via 11 steps using a combination of our previous and new syntheses, and the overall yield for **11** via this new synthesis is improved by 44% with respect to that by our previous synthesis.

## Conclusions

In summary, we have prepared a number of functionalized methallyl halides via CM of methallyl halides as promoted by the Hoveyda–Grubbs second-generation catalyst (**III**) and the Stewart–Grubbs catalyst (**IV**) in moderate to good yield with moderate *E*/*Z* selectivity. In most instances of methallyl halides CM, **IV** was superior to **III**. Several nitrogen-containing functional groups tolerated the CM of methallyl halides when **IV** was employed. Unlike the catalyst **III**, **IV** also efficiently catalyzed the methallyl halide CM of an olefin bearing an *N,N*-dimethyl amide group.

The practicality of this method was demonstrated by accomplishing a formal total synthesis of (–)-presphaerene (**1**). Accordingly, we believe that the CM of methallyl halide represents an excellent and practical alternative to general olefination–reduction–halogenation and Grignard addition–halogenation of aldehydes for the incorporation of methallyl halide groups in natural product synthesis.

## Data Availability Statement

All datasets presented in this study are included in the article/Supplementary Material.

## Author Contributions

JL designed the experiments and supervised the research. SM, JK, and HN carried out the synthesis. SM and JH performed the preparative-LC purification and spectroscopic analyses. HP and JL offered guidance on the project. The manuscript was written by SM, HP, and JL and the final version was edited and approved by all the contributing authors.

## Conflict of Interest

The authors declare that the research was conducted in the absence of any commercial or financial relationships that could be construed as a potential conflict of interest.
