# Supplementary material for: Cross-Metathesis of Methallyl Halides: Concise Enantioselective Formal Total Synthesis of (–)-Presphaerene
Source: Front Chem. 2020 Jun 30;8:494. doi: 10.3389/fchem.2020.00494 (PMC7344242; doi:10.3389/fchem.2020.00494)
Supplement: Supplementary file 1 [file Data_Sheet_1.docx]

Supplementary Material

# Supplementary Tables

**TABLE S1** Cross-metathesis (CM) of methallyl bromide with functionalized olefins.^a^

| Entry | Compound | R– | n | Reaction  time (h) | Yield  (%)^b^ | Ratio  (*E*/*Z*)^c^ |
| --- | --- | --- | --- | --- | --- | --- |
| 1 | **4a** | Me– | 8 | 24 | **15a**, 72 | 1.7:1 |
| 2 | **4b** | AcO– | 2 | 18 | **15b**, 67 | 3.6:1 |
| 3 | **4c** | EtO_2_C– | 1 | 24 | **15c**, 71 | 4.1:1 |
| 4 | **4d** | Ac– | 1 | 36 | **15d**, 57 | 1.2:1 |
| 5 | **4e** | HO– | 7 | 18 | **15e**, 66 | 2.8:1^d^ |
| 6 | **4f** | TBSO– | 7 | 18 | **15f**, 68 | 1.1:1^d^ |
| 7 | **4g** |  | 3 | 18 | **15g**, 54 | 2.0:1^d^ |
| 8 | **4h** | *p*-MeOPh– | 0 | 24 | **15h**, 67 | 3.4:1 |

*^a^Total 20 mol% (time 0, 10 mol%; time 5 h, 10 mol%) of* ***IV*** *was used to complete the reaction. ^b^Isolated yield.*

*^c^The ratio was determined by the analysis of ^1^H 400 MHz NMR spectra.*

*^d^The ratio was determined by the analysis of ^1^H 600 MHz NMR spectra.*

**TABLE S2** CM of methallyl bromide with olefins bearing nitrogen-containing functional groups.^a^

| Entry | Compound | R– | n | Reaction  time (h) | Yield  (%)^b^ | Ratio  (*E*/*Z*)^c^ |
| --- | --- | --- | --- | --- | --- | --- |
| 1 | **6a** | Me(MeO)N(O)C– | 3 | 24 | **16a**, 71 | 4.1:1^d^ |
| 2 | **6b** | Me_2_N(O)C– | 3 | 36 | **16b**, 57 | 1.2:1 |
| 3 | **6c** |  | 2 | 18 | **16c**, 67 | 3.6:1 |
| 4 | **6d** | BocNH– | 2 | 18 | **16d**, 25 | 2.8:1 |
| 5 | **6e** | NC– | 3 | 24 | **16e**, 25 | 8.0:1^d^ |

*^a^Total 20 mol% (time 0, 10 mol%; time 5 h, 10 mol%) of* ***IV*** *was used to complete the reaction.*

*^b^Isolated yield.*

*^c^The ratio was determined by the analysis of ^1^H 400 MHz NMR spectra.*

*^d^The ratio was determined by the analysis of ^1^H 600 MHz NMR spectra.*

**TABLE S3** Cross-metathesis of methallyl bromide with olefin **12**.^a^

| Entry | solvent | product | Yield^b^ [%,(*E*/*Z*)^c^]  **III^d^** **IV^d^** | |
| --- | --- | --- | --- | --- |
| 1 | CH_2_Cl_2_ | **10** | 25 (2.3:1) | 28 (2.3:1) |
| 2 | C_2_H_4_Cl_2_ | **10** | 23 (1.8:1) | 24 (1.9:1) |
| 3 | EtOAc | **10** | 40 (2.5:1) | 42 (2.8:1) |
| 4 | THF | **10** | 25 (3.5:1) | 26 (4.0:1) |
| 5 | benzene | **10** | 42 (2.6:1) | 52 (2.8:1) |
| 6 | toluene | **10** | 48 (2.3:1) | 70 (2.7:1) |

*^a^All reactions were performed with olefin* ***2*** *(0.2 mmol) in solvents (0.1 M) for 24 h at 40 °C under an argon atmosphere.*

*^b^Isolated yield.*

*^c^The ratio was determined by the analysis of ^1^H 400 MHz NMR spectra.*

*^d^Total 20 mol% (time 0, 10 mol%; time 5 h, 10 mol%) of* ***III*** *or* ***IV*** *was used to complete the reaction.*

# Experimental Procedures

**2.1 General experimental**

Proton (^1^H) and carbon (^13^C) NMR spectra were obtained on a Bruker Avance II 600 (600/150 MHz) or Jeol JNM-ECZ400S/L1 (400/100 MHz) spectrometer by using deuterochloroform (CDCl_3_) as the solvent. Chemical shifts (δ) are reported in parts per million (ppm) units with Me_4_Si and the residual CHCl_3_ of deuterochloroform as the internal standard. High-resolution mass spectrometry (HRMS) was carried out on a Jeol JMS-700 mass spectrometer coupled with an Agilent 7890 gas chromatography (GC) system. All reactions were routinely performed under a nitrogen or argon atmosphere. Reactions were monitored by thin-layer chromatography (Kieselgel 60 F254, Merck). Spots were detected by viewing under a UV light and by colorizing via charring after dipping in a mixture of *p*-anisaldehyde, acetic acid, sulfuric acid, and methanol. In the aqueous work-up, all organic solutions were dried over anhydrous MgSO_4_ and filtered prior to rotary evaporation at reduced pressure. The crude products were purified by flash column chromatography on silica gel (ZEOprep 60, 40−63 μm, Zeochem). Unless otherwise stated, materials and solvents were obtained from commercial suppliers and were used without purification. THF was freshly distilled from sodium and benzophenone, and methylene chloride was purified by refluxing with P_2_O_5_.

**2.2 Typical procedure for cross-metathesis (CM) of methallyl halides**

To a solution of olefin **2** (50 mg, 0.28 mmol) in anhydrous toluene (degassed, 2.8 mL) in a sealed tube, methallyl chloride (0.11 mL, 1.12 mmol) and Stewart-Grubbs catalyst **IV** (16 mg, 0.028 mmol) were added at room temperature. The reaction mixture was stirred for 5 h at 40 °C. Methallyl chloride (0.11 mL, 1.12 mmol) and ruthenium complex **IV** (16 mg, 0.028 mmol) were then additionally added. The mixture was stirred at 40 °C for 13 h and then concentrated at reduced pressure. The resulting residue was purified by flash column chromatography on silica gel (hexanes/ether, 99:1) to afford **3a** (53 mg, colorless oil, *E/Z* = 3.3:1 by analysis of the ^1^H 400 MHz NMR spectrum) as an inseparable *E/Z* mixture in 78% yield.

**2.2.1 (*E/Z*)-(((6-Chloro-5-methylhex-4-en-1-yl)oxy)methyl)benzene (3a)**

Colorless oil, 78% yield. ^1^H NMR (400 MHz, CDCl_3_) δ 7.38–7.31 (m, 4H), 7.31–7.26 (m, 1H), [5.52 (t, *J* = 6.9 Hz) and 5.37 (ddd, *J* = 7.5, 7.5, 1.2 Hz), 1H], 4.50 (s, 2H), [4.06 (s) and 4.01 (s), 2H], 3.47 (t, *J* = 6.4 Hz, 2H), 2.20–2.10 (m, 2H), [1.82 (d, *J* = 1.2 Hz) and 1.73 (s), 3H], 1.72–1.64 (m, 2H); ^13^C NMR (100 MHz, CDCl_3_) δ 138.5, 132.2, 130.3, 128.3, 127.6, 127.5, 72.9, 69.5, 52.4, 29.2, 24.7, 24.5, 14.1 (peaks of the *Z*-isomer detected separately from those of the *E*-isomer δ 131.9, 130.5, 127.7, 69.4, 43.6, 29.5, 24.5, 21.5); HRMS (EI) *m*/*z* calcd for C_14_H_19_ClO (M^+^) 238.1124, found 238.1126.

**2.2.2 (*E/Z*)-(((6-Bromo-5-methylhex-4-en-1-yl)oxy)methyl)benzene (3b)**

Colorless oil, 68% yield. ^1^H NMR (400 MHz, CDCl_3_) δ 7.39–7.31 (m, 4H), 7.31–7.26 (m, 1H), [5.59 (t, *J* = 7.1 Hz), 5.38 (ddd, *J* = 7.5, 7.5, 1.3 Hz), 1H], [4.50 (s), 4.49 (s), 2H], [3.98 (s), 3.96 (s), 2H], [3.48 (t, *J* = 6.3 Hz) and 3.47 (t, *J* = 6.3 Hz), 2H], 2.20–2.09 (m, 2H), [1.83 (d, *J* = 1.2 Hz) and 1.75 (s), 3H] 1.74–1.64 (m, 2H); ^13^C NMR (150 MHz, CDCl_3_) δ 138.5, 132.5, 130.8, 128.3, 127.6, 72.9, 69.5, 41.6, 29.1, 24.9, 14.6 (peaks of the *Z*-isomer detected separately from those of the *E*-isomer δ 132.1, 131.0, 127.5, 32.2, 29.3, 24.7, 21.8); HRMS (EI) *m*/*z* calcd for C_14_H_19_BrO (M^+^) 282.0619, found 282.0614.

**2.2.3 (*E/Z*)-1-Chloro-2-methyltridec-2-ene (5a)**

Colorless oil, 80% yield. ^1^H NMR (400 MHz, CDCl_3_) δ [5.53 (t, *J* = 6.8 Hz) and 5.39 (ddd, *J* = 7.5, 7.5, 1.3 Hz), 1H], [4.07 (s) and 4.02 (s), 2H], 2.10–1.98 (m, 2H), [1.82 (d, *J* = 1.5 Hz) and 1.73 (s), 3H], 1.41–1.30 (m, 2H), 1.26 (s, 14H), 0.88 (t, *J* = 6.8 Hz, 3H); ^13^C NMR (100 MHz, CDCl_3_) δ 131.4, 131.3, 52.7, 31.9, 29.6, 29.5, 29.3, 29.3, 29.2, 28.0, 22.7, 14.1, 14.0 (peaks of the *Z*-isomer detected separately from those of the *E*-isomer δ 131.6, 131.0, 43.8, 29.2, 27.9, 21.5); HRMS (EI) *m*/*z* calcd for C_14_H_27_Cl (M^+^) 230.1801, found 230.1801.

**2.2.4 (*E/Z*)-1-Bromo-2-methyltridec-2-ene (15a)**

Pale yellow oil, 72% yield. ^1^H NMR (400 MHz, CDCl_3_) δ [5.60 (t, *J* = 7.2 Hz) and 5.39 (ddd, *J* = 7.4, 7.4, 1.3 Hz), 1H], [3.99 (s) and 3.98 (s), 2H], 2.01 (dd, *J* = 14.4, 7.2 Hz, 3H), [1.83 (dd, *J* = 3.0, 1.8 Hz) and 1.74 (dd, *J* = 3.3, 2.9 Hz), 3H], 1.40–1.30 (m, 2H), 1.26 (s, 14H), 0.88 (t, *J* = 6.8 Hz, 3H); ^13^C NMR (150 MHz, CDCl_3_) δ 131.9, 131.8, 42.0, 31.9, 29.6, 29.5, 29.3, 29.3, 29.1, 28.3, 22. 7, 14.6, 14.1 (peaks of the *Z*-isomer detected separately from those of the *E*-isomer δ 132.1, 37.2, 29.5, 29.4, 28.9, 27.9, 21.9); HRMS (EI) *m*/*z* calcd for C_14_H_27_Br (M^+^) 274.1296, found 274.1294.

**2.2.5 (*E/Z*)-6-Chloro-5-methylhex-4-en-1-yl acetate (5b)**

Colorless oil, 81% yield. ^1^H NMR (400 MHz, CDCl_3_) δ [5.51 (t, *J* = 6.9 Hz) and 5.36 (t, *J* = 8.2 Hz) 1H], 4.05 (t, *J* = 6.6 Hz, 2H), 4.00 (s, 2H), 2.20–207 (m, 2H), [2.05 (s) and 2.04 (s), 3H], [1.82 (d, *J* = 1.2 Hz) and 1.73 (s) 3H], 1.72–1.65 (m, 2H); ^13^C NMR (100 MHz, CDCl_3_) δ 171.06, 132.7, 129.3, 63.7, 52.1, 28.0, 24.4, 20.9, 14.1 (peaks of the *Z*-isomer detected separately from those of the *E*-isomer δ 132.4, 129.7, 63.6, 43.3, 28.3, 24.2, 21.5). HRMS (EI) *m*/*z* calcd for C_9_H_15_O_2_ (M^+^ – Cl) 155.1067, found 155.1077.

**2.2.6 (*E/Z*)-6-Bromo-5-methylhex-4-en-1-yl acetate (15b)**

Colorless oil, 67% yield. ^1^H NMR (400 MHz, CDCl_3_) δ [5.58 (t, *J* = 7.2 Hz) and 5.36 (ddd, *J* = 7.5, 7.5, 1.2 Hz), 1H], 4.05 (dd, *J* = 12.5, 6.1 Hz, 2H), 3.95 (s, 2H), 2.18–2.06 (m, 2H), [2.05 (s) and 2.04 (s), 3H], [1.83 (d, *J* = 1.1 Hz) and 1.75 (s), 3H], 1.74–1.65 (m, 3H); ^13^C NMR (100 MHz, CDCl_3_) δ 171.1, 133.0, 129.8, 63.7, 41.3, 27.9, 24.6, 20.9, 14.6 (peaks of the *Z*-isomer detected separately from those of the *E*-isomer δ 132.6, 130.0, 63.6, 31.8, 28.1, 24.4, 21.9); HRMS (EI) *m*/*z* calcd for C_9_H_15_BrO_2_ (M^+^) 234.0255, found 234.0258.

**2.2.7 (*E/Z*)-Ethyl 6-chloro-5-methylhex-4-enoate (5c)**

Colorless oil, 83% yield. ^1^H NMR (400 MHz, CDCl_3_) δ [5.50 (t, *J* = 6.1 Hz) and 5.38–5.31 (m) 1H], 4.12 (q, *J* = 7.1 Hz, 2H), [4.07 (s), and 3.99 (s), 2H], 2.42–2.31 (m, 4H), [1.81 (d, *J* = 0.9 Hz), and 1.75 (s), 3H], 1.24 (t, *J* = 7.1 Hz, 3H); ^13^C NMR (100 MHz, CDCl_3_) δ 172.9, 133.1, 132.8, 128.8, 128.5, 60.4, 52.0, 43.3, 34.0, 33.7, 23.5, 23.3, 21.6, 14.2, 14.1 (peaks of the *Z*-isomer detected separately from those of the *E*-isomer δ 132.6, 130.0, 63.6, 31.8, 28.1, 24.4, 21.9); HRMS (EI) *m*/*z* calcd for C_9_H_15_ClO_2_ (M^+^) 190.0761, found 190.0762.

**2.2.8 (*E/Z*)-Ethyl 6-bromo-5-methylhex-4-enoate (15c)**

Colorless oil, 71% yield. ^1^H NMR (400 MHz, CDCl_3_) δ [5.58 (t, *J* = 7.2 Hz) and 5.35 (t, *J* = 6.5 Hz), 1H], 4.13 (q, *J* = 7.1 Hz, 2H), [3.98 (s) and 3.94 (s), 2H], [2.38 (d, *J* = 3.1 Hz) and 2.35 (d, *J* = 3.2 Hz), 4H], [1.83 (s) and 1.77 (s), 3H], 1.25 (t, *J* = 7.1 Hz, 3H); ^13^C NMR (100 MHz, CDCl_3_) δ 172.84, 133.42, 133.07, 129.17, 128.98, 60.42, 41.10, 33.65, 33.56, 31.70, 23.76, 23.45, 21.90, 14.65, 14.21 (peaks of the *Z*-isomer detected separately from those of the *E*-isomer δ 132.6, 130.0, 63.6, 31.8, 28.1, 24.4, 21.9); HRMS (EI) *m*/*z* calcd for C_9_H_15_BrO_2_ (M^+^) 234.0255, found 234.0252.

**2.2.9 (*E/Z*)-7-Chloro-6-methylhept-5-en-2-one (5d)**

Pale-yellow oil, 77% yield. ^1^H NMR (400 MHz, CDCl_3_) δ [5.49 (t, *J* = 6.9 Hz), and 5.34 (ddd, *J* = 7.5, 7.5, 1.1 Hz), 1H], [4.08 (s) and 3.99 (s) 2H], 2.51 (t, *J* = 7.1 Hz, 2H), 2.37–2.26 (m, 2H), 2.14 (s, 3H), [1.81 (d, *J* = 1.1 Hz) and 1.75 (s), 3H]; ^13^C NMR (100 MHz, CDCl_3_) δ 207.9, 132.8, 128.8, 52.1, 42.74, 29.93, 22.2, 14.1 (peaks of the *Z*-isomer detected separately from those of the *E*-isomer δ 207.8, 132.6, 129.2, 43.33, 43.07, 22.0, 21.6); HRMS (EI) *m*/*z* calcd for C_8_H_13_ClO (M^+^) 160.0655, found 160.0651.

**2.2.10 (*E/Z*)-7-Bromo-6-methylhept-5-en-2-one (15d)**

Pale-brown oil, 57% yield. ^1^H NMR (400 MHz, CDCl_3_) δ [5.54 (t, *J* = 7.2 Hz) and 5.33 (ddd, *J* = 7.5, 7.5, 1.4 Hz), 1H], [3.98 (s) and 3.94 (s), 2H], 2.56–2.45 (m, 2H), 2.38–2.24 (m, 2H), [2.14 (s) and 2.14 (s), 3H], [1.82 (dd, *J* = 2.5, 1.2 Hz) and 1.77 (s), 3H]; ^13^C NMR (150 MHz, CDCl_3_) δ 207.9, 133.2, 129.3, 42.6, 41.2, 30.0, 22.5, 14.6 (peaks of the *Z*-isomer detected separately from those of the *E*-isomer δ 207.8, 132.8, 129.5, 42.7, 31.8, 22.0, 21.9); HRMS (EI) *m*/*z* calcd for C_8_H_13_BrO (M^+^) 204.0150, found 204.0155.

**2.2.11 (*E/Z*)-11-chloro-10-methylundec-9-en-1-ol (5e)**

Pale-yellow oil, 75% yield. ^1^H NMR (400 MHz, CDCl_3_) δ [5.53 (t, *J* = 7.1 Hz) and 5.38 (t, *J* = 7.5 Hz), 1H], [4.07 (s) and 4.02 (s), 2H], 3.64 (t, *J* = 6.5 Hz, 2H), 2.14–197 (m, 2H), [1.82 (s) and 1.73 (s), 3H], 1.61–1.50 (m, 2H), 1.43–1.21 (m, 10H); ^13^C NMR (150 MHz, CDCl_3_) δ 131.5, 131.2, 63.0, 52.6, 32.7, 29.4, 29.3, 29.1, 29.1, 28.0, 25.7, 14.1 (peaks of the *Z*-isomer detected separately from those of the *E*-isomer δ 43.8, 29.5, 27.8, 21.5); HRMS (EI) *m*/*z* calcd for C_12_H_23_ClO (M^+^) 218.1437, found 218.1432.

**2.2.12 (*E/Z*)-11-Bromo-10-methylundec-9-en-1-ol (15e)**

Pale-yellow oil, 66% yield. ^1^H NMR (600 MHz, CDCl_3_) δ [5.59 (t, *J* = 7.2 Hz) and 5.39 (ddd, *J* = 7.5, 7.5, 1.4 Hz), 1H], [3.98 (s) and 3.97 (s), 2H], 3.64 (t, *J* = 6.6 Hz, 2H), 2.07–197 (m, 2H), 1.74 (s, 3H), 1.61–1.52 (m, 2H), 1.40–1.24 (m, 10H); ^13^C NMR (150 MHz, CDCl_3_) δ 132.0, 131.8, 63.0, 42.0, 32.7, 32.5, 29.4, 29.3, 29.1, 29.1, 28.2, 25.7, 14.6 (peaks of the *Z*-isomer detected separately from those of the *E*-isomer δ 37.4, 29.3, 28.8, 21.8); HRMS (EI) *m*/*z* calcd for C_12_H_23_BrO (M^+^) 262.0932, found 262.0936.

**2.2.13 (*E/Z*)-*tert*-Butyl((11-chloro-10-methylundec-9-en-1-yl)oxy)dimethylsilane (5f)**

Pale-yellow oil, 81% yield. ^1^H NMR (400 MHz, CDCl_3_) δ [5.53 (t, *J* = 7.1 Hz) and 5.38 (t, *J* = 7.4 Hz), 1H], [4.06 (s) and 4.02 (s), 2H], 3.59 (t, *J* = 6.6 Hz, 2H), 2.10–1.98 (m, 2H), [1.82 (d, *J* = 1.0 Hz) and 1.73 (s), 3H], 1.55–1.45 (m, 2H), 1.40–1.30 (m, 2H), 1.29 (s, 8H), 0.89 (s, 9H), 0.05 (s, 6H); ^13^C NMR (100 MHz, CDCl_3_) δ 131.5, 131.3, 63.3, 52.6, 32.9, 29.5, 29.4, 29.2, 29.1, 28.0, 26.0, 25.8, 18.4, 14.1, -5.3 (peaks of the *Z*-isomer detected separately from those of the *E*-isomer δ 131.6, 131.1, 43.8, 29.6, 29.2, 27.9, 21.5); HRMS (EI) *m*/*z* calcd for C_18_H_37_ClOSi (M^+^) 332.2302, found 332.2305.

**2.2.14 (*E/Z*)-*tert*-Butyl((11-bromo-10-methylundec-9-en-1-yl)oxy)dimethylsilane (15f)**

Pale-yellow oil, 68% yield. ^1^H NMR (600 MHz, CDCl_3_) δ [5.60 (t, *J* = 7.1 Hz) and 5.39 (ddd, *J* = 7.4, 7.4, 1.3 Hz), 1H], [3.98 (s) and 3.98 (s) 2H], 3.59 (t, *J* = 6.6 Hz, 2H), [2.08–2.03 (m) and 2.01 (dd, *J* = 14.6, 7.3 Hz), 2H], [1.83 (d, *J* = 1.1 Hz) and 1.75 (s), 3H], 1.54−145 (m, 2H), 1.40–130 (m, 2H), 1.29 (s, 8H), 0.89 (s, 9H), 0.04 (s, 6H); ^13^C NMR (150 MHz, CDCl_3_) δ 131.8, 131.7, 63.3, 42.0, 32.9, 29.5, 29.4, 29.2, 29.0, 28.3, 26.0, 25.8, 18.4, 14.6, -5.3 (peaks of *Z*-isomer detected separately from those of *E*-isomer δ 132.0, 37.2, 32.5, 29.3, 28.1, 21.9); HRMS (EI) *m*/*z* calcd for C_18_H_37_BrOSi (M^+^) 376.1797, found 376.1794.

**2.2.15 (*E/Z*)-2-(7-Chloro-6-methylhept-5-en-1-yl)oxirane (5g)**

Pal-yellow oil, 83% yield. ^1^H NMR (600 MHz, CDCl_3_) δ [5.52 (t, *J* = 7.0 Hz) and 5.42–5.33 (m), 1H], [4.06 (s) and 4.01 (s), 2H], 2.93–2.86 (m, 1H), 2.74 (t, *J* = 4.6 Hz, 1H), 2.46 (dd, *J* = 5.0, 2.7 Hz, 1H), 2.12–202 (m, 2H), [1.82 (d, *J* = 1.1 Hz) and 1.73 (s), 3H], 1.58–1.37 (m, 6H); ^13^C NMR (150 MHz, CDCl_3_) δ 131.9, 130.7, 52.5, 52.2, 47.1, 32.3, 28.9, 27.9, 25.6, 14.1 (peaks of the *Z*-isomer detected separately from those of the *E*-isomer δ 131.5, 131.0, 43.6, 32.3, 29.3, 27.7, 25.5, 21.5); HRMS (EI) *m*/*z* calcd for C_10_H_17_ClO (M^+^) 188.0968, found 188.0964.

**2.2.16 (*E/Z*)-2-(7-Bromo-6-methylhept-5-en-1-yl)oxirane (15g)**

Pale-yellow oil, 54% yield. ^1^H NMR (600 MHz, CDCl_3_) δ [5.60 (t, *J* = 7.3 Hz) and 5.39 (t, *J* = 7.6 Hz), 1H], 3.98 (s) and 3.97 (s, 2H), 2.90 (s, 1H), 2.75 (t, *J* = 4.4 Hz, 1H), 2.46 (dd, *J* = 4.7, 2.7 Hz, 1H), 2.13–2.00 (m, 2H), [1.84 (d, *J* = 6.2 Hz) and 1.75 (s), 3H], 1.55–1.37 (m, 6H); ^13^C NMR (150 MHz, CDCl_3_) δ 132.2, 131.2, 52.2, 47.1, 41.8, 32.3, 28.8, 28.1, 25.6, 21.9, 14.6 (peaks of the *Z*-isomer detected separately from those of the *E*-isomer δ 131.4, 29.0, 27.9, 21.9); HRMS (EI) *m*/*z* calcd for C_10_H_17_BrO (M^+^) 232.0463, found 232.0466.

**2.2.17 (*E/Z*)-1-(4-Chloro-3-methylbut-2-en-1-yl)-4-methoxybenzene (5h)**

Pale-yellow oil, 77% yield. ^1^H NMR (400 MHz, CDCl_3_) δ [7.12 (s) and 7.09 (d, *J* = 8.4 Hz), 2H], 6.85 (d, *J* = 8.6 Hz, 2H), [5.71 (t, *J* = 7.3 Hz) and 5.60–5.49 (m), 1H], [4.17 (s) and 4.06 (s), 2H], 3.79 (s, 3H), [3.38 (d, *J* = 7.9 Hz) and 3.35 (d, *J* = 7.4 Hz), 2H], [1.88 (d, *J* = 1.7 Hz) and 1.86 (s), 3H]; ^13^C NMR (100 MHz, CDCl_3_) δ 158.0, 132.3, 132.2, 129.7, 129.2, 113.9, 55.3, 52.2, 33.3, 14.2 (peaks of the *Z*-isomer detected separately from those of the *E*-isomer δ 158.1, 132.1, 131.8, 129.9, 129.3, 43.5, 33.3, 21.6); HRMS (EI) *m*/*z* calcd for C_12_H_15_ClO (M^+^) 210.0811, found 210.0814.

**2.2.18 (*E/Z*)-1-(4-Bromo-3-methylbut-2-en-1-yl)-4-methoxybenzene (15h)**

Pale-yellow oil, 67% yield. ^1^H NMR (400 MHz, CDCl_3_) δ [7.12 (d, *J* = 8.7 Hz) and 7.08 (d, *J* = 8.7 Hz), 2H], 6.84 (d, *J* = 8.6 Hz, 2H), [5.78 (t, *J* = 7.3 Hz) and 5.55 (ddd, *J* = 7.6, 7.6, 1.3 Hz), 1H], [4.08 (s) and 4.01 (s), 2H], 3.79 (s, 3H), [3.37 (d, *J* = 8.1 Hz) and 3.34 (d, *J* = 7.3 Hz), 2H], [1.89 (d, *J* = 1.2 Hz) and 1.88 (s), 3H]; ^13^C NMR (100 MHz, CDCl_3_) δ 158.0, 132.6, 132.1, 130.1, 129.2, 114.0, 55.3, 41.4, 33.6, 14.8 (peaks of the *Z*-isomer detected separately from those of the *E*-isomer δ 132.0, 131.9, 130.3, 129.3, 33.4, 32.0, 21.9); HRMS (EI) *m*/*z* calcd for C_12_H_15_BrO (M^+^) 254.0306, found 254.0303.

**2.2.19 (*E/Z*)-8-Chloro-*N*-methoxy-*N*,7-dimethyloct-6-enamide (7a)**

Colorless oil, 83% yield. ^1^H NMR (600 MHz, CDCl_3_) δ [5.52 (t, *J* = 7.0 Hz) and 5.38 (t, *J* = 6.9 Hz), 1H], [4.05 (s) and 4.00 (s), 2H], 3.67 (s, 3H), 3.17 (s, 3H), 2.41 (t, *J* = 7.4 Hz, 2H), 2.13–2.02 (m, 2H), [1.81 (d, *J* = 1.2 Hz) and 1.73 (s), 3H], 1.67–1.60 (m, 2H), 1.45–1.37 (m, 2H); ^13^C NMR (150 MHz, CDCl_3_) δ 174.5, 131.9, 130.6, 61.2, 52.5, 32.1, 31.7, 28.9, 27.8, 24.2, 14.1 (Peaks of *Z*-isomer detected separately from those of *E*-isomer δ 131.5, 131.0, 43.7, 29.3, 27.6, 24.2 and 21.5); HRMS (EI) *m*/*z* calcd for C_11_H_20_BrNO_2_ (M^+^) 233.1183, found 233.1180.

**2.2.20 (*E/Z*)-8-bromo-*N*-methoxy-*N*,7-dimethyloct-6-enamide (16a)**

Pale-yellow oil, 71% yield. ^1^H NMR (600 MHz, CDCl_3_) δ [5.60 (t, *J* = 7.1 Hz) and 5.39 (t, *J* = 6.8 Hz), 1H], [3.98 (s) and 3.96 (s), 2H], [3.68 (s) and 3.68 (s), 3H], 3.17 (s, 3H), 2.41 (t, *J* = 6.8 Hz, 2H), 2.13–2.02 (m, 2H), [1.82 (d, *J* = 1.2 Hz) and 1.75 (s), 3H], 1.70–1.58 (m, 2H), 1.48–1.37 (m, 2H); ^13^C NMR (150 MHz, CDCl_3_) δ 174.5, 132.2, 131.2, 61.2, 41.8, 32.4, 31.7, 28.8, 28.1, 24.2, 14.6 (peaks of the *Z*-isomer detected separately from those of the *E*-isomer δ 131.7, 131.4, 32.1, 29.0, 27.8, 21.9); HRMS (EI) *m*/*z* calcd for C_11_H_20_BrNO_2_ (M^+^) 277.0677, found 277.0674.

**2.2.21 (*E/Z*)-8-Chloro-*N*,*N*,7-trimethyloct-6-enamide (7b)**

Colorless oil, 77% yield. ^1^H NMR (400 MHz, CDCl_3_) δ [5.53 (t, *J* = 6.9 Hz) and 5.38 (t, *J* = 7.5, 6.2 Hz), 1H], [4.06 (s) and 4.01 (s), 2H], 2.99 (s, 3H), 2.93 (s, 3H), 2.29 (t, *J* = 7.3 Hz, 2H), 2.14–202 (m, 2H), [1.81 (t, *J* = 1.3 Hz) and 1.73 (s), 3H], 1.69–1.59 (m, 2H), 1.47–1.36 (m, 2H); ^13^C NMR (100 MHz, CDCl_3_) δ 172.9, 131.9, 130.7, 52.5, 37.2, 35.3, 33.2, 29.0, 27.8, 24.7, 14.1 (peaks of the *Z*-isomer detected separately from those of the *E*-isomer δ 131.5, 131.0, 43.7, 33.1, 29.3, 27.7, 24.7, 21.5); HRMS (EI) *m*/*z* calcd for C_11_H_20_ClNO (M^+^) 217.1233, found 217.1237.

**2.2.22 (*E/Z*)-8-Bromo-*N*,*N*,7-trimethyloct-6-enamide (16b)**

Colorless oil, 57% yield. ^1^H NMR (400 MHz, CDCl_3_) δ 5.59 (t, *J* = 7.2 Hz) and 5.39 (ddd, *J* = 7.4, 7.4, 1.3 Hz), 1H], [3.97 (s) and 3.96 (s), 2H], 2.99 (s, 3H), 2.93 (s, 3H), 2.35–2.25 (m, 2H), 2.13–2.01 (m, 2H), [1.82 (d, *J* = 1.3 Hz) and 1.74 (s), 3H], 1.71–1.58 (m, 2H), 1.49–1.35 (m, 2H); ^13^C NMR (100 MHz, CDCl_3_) δ 172.9, 132.2, 131.2, 41.8, 37.3, 35.4, 33.2, 28.9, 28.1, 24.7, 14.6 (peaks of the *Z*-isomer detected separately from those of the *E*-isomer δ 131.7, 131.4, 33.1, 32.4, 29.0, 27.8, 24.7, 21.8); HRMS (EI) *m*/*z* calcd for C_11_H_20_BrNO (M^+^) 261.0728, found 261.0725.

**2.2.23 (*E/Z*)-2-(6-Chloro-5-methylhex-4-en-1-yl)isoindoline-1,3-dione (7c)**

Colorless oil, 81% yield. ^1^H NMR (400 MHz, CDCl_3_) δ 7.83 (dd, *J* = 5.3, 3.1 Hz, 2H), 7.70 (dd, *J* = 5.5, 3.0 Hz, 2H), [5.52 (t, *J* = 6.8 Hz) and 5.36 (ddd, *J* = 7.4, 7.4, 1.3 Hz), 1H], [4.03 (s) and 3.95 (s) 2H], 3.68 (t, *J* = 7.2 Hz, 2H), 2.18–2.05 (m, 2H), 1.82–1.74 (m, 2H), 1.71 (s, 3H); ^13^C NMR (150 MHz, CDCl_3_) δ 168.3, 133.9, 132.1, 129.3, 123.2, 52.1, 37.6, 27.9, 25.5, 21.5, 14.2 (peaks of the *Z*-isomer detected separately from those of the *E*-isomer δ 132.7, 129.7, 43.4, 28.2, 25.3, 21.5; HRMS (EI) *m*/*z* calcd for C_15_H_16_ClNO_2_ (M^+^) 277.0870, found 277.0872.

**2.2.24 (*E/Z*)-2-(6-Bromo-5-methylhex-4-en-1-yl)isoindoline-1,3-dione (16c)**

Colorless oil, 67% yield. ^1^H NMR (400 MHz, CDCl_3_) δ 7.84 (dd, *J* = 5.4, 3.1 Hz, 2H), 7.71 (dd, *J* = 5.5, 3.0 Hz, 2H), [5.60 (t, *J* = 7.0 Hz) and 5.39 (ddd, *J* = 7.4, 7.4, 1.3 Hz) 1H], [3.96 (s) and 3.92 (s) 2H], 3.74–3.65 (m, 2H), 2.17–2.03 (m, 2H), 1.79 (dd, *J* = 4.3, 3.1 Hz, 2H), [1.76 (s) and 1.74 (s) 3H]; ^13^C NMR (150 MHz, CDCl_3_) δ 168.3, 133.9, 132.1, 129.8, 123.2, 41.2, 37.6, 27.8, 25.7, 14.7 (peaks of the *Z*-isomer detected separately from those of the *E*-isomer δ 133.1, 130.1, 31.9, 28.0, 25.4, 21.9); HRMS (EI) *m*/*z* calcd for C_15_H_16_BrNO_2_ (M^+^) 321.0364, found 321.0367.

**2.2.25 (*E/Z*)-*tert*-Butyl (6-chloro-5-methylhex-4-en-1-yl)carbamate (7d)**

Pale-yellow oil, 63% yield. ^1^H NMR (400 MHz, CDCl_3_) δ [5.51 (t, *J* = 7.1 Hz) and 5.36 (t, *J* = 7.0 Hz) 1H], 4.62–4.44 (m, 1H), [4.05 (s) and 4.00 (s), 2H], 3.11 (q, *J* = 6.8 Hz, 2H), 2.15–2.02 (m, 2H), [1.82 (d, *J* = 1.0 Hz) and 1.73 (s), 3H], 1.61–1.50 (m, 2H), 1.44 (s, 9H); ^13^C NMR (100 MHz, CDCl_3_) δ 155.9, 132.5, 129.8, 79.2, 52.3, 40.2, 29.5, 28.4, 25.3, 14.1 (peaks of the *Z*-isomer detected separately from those of the *E*-isomer δ 132.1, 130.2, 43.5, 29.8, 25.1, 21.6; HRMS (EI) *m*/*z* calcd for C_12_H_22_ClNO_2_ (M^+^) 247.1339, found 247.1335.

**2.2.26 (*E/Z*)-*tert*-Butyl (6-bromo-5-methylhex-4-en-1-yl)carbamate (16d)**

Pale-yellow oil, 25% yield. Compound **16d** was not stable and some portion of the purified product was decomposed during the course of NMR experiments. We analyzed only the identifiable peaks in the NMR spectra of compound **16d**. ^1^H NMR (400 MHz, CDCl_3_) δ [5.59 (t, *J* = 7.1 Hz) and 5.37 (t, *J* = 7.3 Hz), 1H], 4.62–4.44 (m, 1H), [3.97 (s) and 3.96 (s), 2H], 3.11 (d, *J* = 6.8 Hz, 2H), 2.14–2.01 (m, 2H), [1.83 (d, *J* = 1.3 Hz) and 1.75 (s), 3H], 1.62–1.51 (m, 2H), 1.45 (s, 9H); ^13^C NMR (100 MHz, CDCl_3_) δ 156.0, 132.8, 130.3, 79.2, 41.5, 40.2, 29. 4, 28.4, 25.6, 14.6; HRMS (ESI) *m*/*z* calcd for C_12_H_23_BrNO_2_ (MH^+^) 292.0912, found 292.0909.

**2.2.27 (*E/Z*)-8-Chloro-7-methyloct-6-enenitrile (7e)**

Pale-yellow oil, 79% yield. ^1^H NMR (600 MHz, CDCl_3_) δ [5.50 (t, *J* = 7.0 Hz) and 5.34 (ddd, *J* = 7.5, 7.5, 1.1 Hz), 1H], [4.04 (s) and 4.00 (s), 2H], 2.34 (ddd, *J* = 10.9, 7.2, 3.8 Hz, 2H), 2.15–2.05 (m, 2H), [1.82 (d, *J* = 1.2 Hz) and 1.73 (s), 3H], 1.69–1.63 (m, 2H), 1.58–1.50 (m, 2H); ^13^C NMR (150 MHz, CDCl_3_) δ 132.7, 129.4, 119.5, 52.1, 28.1, 27.1, 24.8, 17.0, 14.1 (peaks of the *Z*-isomer detected separately from those of the *E*-isomer δ 132.4, 129.8, 43.3, 28.3, 26.9, 24.7, 21.6); HRMS (EI) *m*/*z* calcd for C_9_H_14_ClN (M^+^) 171.0815, found 171.0812.

**2.2.28 (*E/Z*)-8-Bromo-7-methyloct-6-enenitrile (16e)**

Pale-yellow oil, 25% yield. ^1^H NMR (600 MHz, CDCl_3_) δ [5.57 (t, *J* = 7.3 Hz) and 5.35 (t, *J* = 7.4 Hz), 1H], [3.96 (s) and 3.96 (s), 2H], 2.35 (t, *J* = 7.1 Hz, 2H), 2.16–2.05 (m, 2H), 1.76 (s, 3H), 1.67 (ddd, *J* = 15.4, 12.6, 7.1 Hz, 2H), 1.60–1.50 (m, 2H); ^13^C NMR (150 MHz, CDCl_3_) δ 133.1, 129.9, 119.5, 41.2, 28.0, 27.4, 24.9, 17.1, 14.7 (peaks of the *Z*-isomer detected separately from those of the *E*-isomer δ 129.4, 27.1). HRMS (EI) *m*/*z* calcd for C_9_H_14_BrN (M^+^) 215.0310, found 215.0313.

**2.2.29 (*S*)-Ethyl 3-(*m*-tolyl)hept-6-enoate (14)**

To a solution of *m*-bromotoluene (2.0 mL, 16.2 mmol) in ether (9.0 mL) was added *n*-BuLi (10.0 mL, 16.2 mmol, 1.6 M in hexanes) at 0 °C. The reaction mixture was stirred for 1 h at room temperature and cooled to -78 °C. Trimethoxyborane (1.84 mL, 16.2 mmol) was added. The mixture was stirred for 30 min at -78 °C, warmed to room temperature, and stirred for 1 h. To the reaction mixture were added water (2.0 mL), 1,4-dioxane (40 mL), Rh(acac)(C_2_H_4_)_4_ (217 mg, 0.84 mmol), and (*R*)-BINAP (646 mg, 1.04 mmol). The mixture was stirred for 10 min. A solution of dienoate **13** (1.00 g, 6.48 mmol) in ether (10 mL) was added and the mixture was stirred for 16 h at 90 °C. The reaction mixture was quenched with saturated aqueous NaHCO_3_ and extracted with ether (3 × 50 mL). The combined organic layers were dried over anhydrous MgSO_4_ and concentrated at reduced pressure. The resulting residue was purified by flash column chromatography on silica gel (hexanes/ether, 99:1) to furnish ester **14** (1.36 g, colorless oil) in 85% yield: [α]^25^_D_ = +7.4 (*c* 0.13, CHCl_3_); ^1^H NMR (600 MHz, CDCl_3_) δ 7.18 (t, *J* = 7.5 Hz, 1H), 7.03–6.97 (m, 3H), 5.82–5.70 (m, 1H), 4.99–4.90 (m, 2H), 4.04 (q, *J* = 7.1 Hz, 2H), 3.12–3.04 (m, 1H), 2.64–2.52 (m, 2H), 2.33 (s, 3H), 1.93–1.89 (m, 2H), 1.78–1.65 (m, 2H), 1.15 (t, *J* = 7.1 Hz, 3H); ^13^C NMR (150 MHz, CDCl_3_) δ 172.4, 143.6, 138.3, 137.9, 128.3, 128.3, 127.2, 124.5, 114.7, 60.2, 41.8, 41.6, 35.2, 31.4, 21.5, 14.1; HRMS (EI) *m*/*z* calcd for C_16_H_22_O_2_ (M^+^) 246.1620, found 246.1620.

**2.2.30 (3*S*)-Ethyl 2-methyl-3-(*m*-tolyl)hept-6-enoate (12)**

To a solution of ester **14** (250 mg, 1.01 mmol) in anhydrous THF (5 mL) was added NaHMDS (2.0 mL, 1.0 M solution in THF) at 0 °C. The reaction mixture was stirred for 0.5 h at the same temperature and iodomethane (0.32 mL, 5.1 mmol) was added. The reaction mixture was stirred for 1 h at 0 °C, warmed to room temperature, and stirred for 24 h. The reaction mixture was quenched with saturated aqueous NH_4_Cl and extracted with ether (3 × 20 mL). The combined organic layers were dried over anhydrous MgSO_4_ and concentrated at reduced pressure. The resulting residue was purified by flash column chromatography on silica gel (hexanes/ether, 99:1) to give a diastereomeric mixture of **12** (major isomer/minor isomer = 3:2, 228 mg, colorless oil) in 86% yield. The two diastereomers of **12** were separated to obtain characterization data. Data for the major diastereomer of **12**: [α]^25^_D_ = −6.6 (*c* 0.53, CHCl_3_); ^1^H NMR (400 MHz, CDCl_3_) δ 7.18 (t, *J* = 7.5 Hz, 1H), 7.02 (d, *J* = 7.4 Hz, 1H), 6.92 (d, *J* = 8.0 Hz, 2H), 5.77–5.65 (m, 1H), 4.90 (dd, *J* = 13.7, 2.0 Hz, 2H), 4.18 (q, *J* = 7.1 Hz, 2H), 2.77–2.61 (m, 1H), 2.65–2.55 (m, 1H), 2.33 (s, 3H), 1.85–1.72 (m, 2H), 1.71–1.62 (m, 2H), 1.29 (t, *J* = 7.1 Hz, 3H), 0.89 (d, *J* = 6.9 Hz, 3H); ^13^C NMR (100 MHz, CDCl_3_) δ 176.5, 141.9, 138.4, 137.9, 129.1, 128.2, 127.3, 125.4, 114.5, 60.3, 48.5, 46.1, 33.6, 31.5, 21.5, 16.2, 14.3; HRMS (EI) *m*/*z* calcd for C_17_H_24_O_2_ (M^+^) 260.1776, found 260.1778. Data for the minor diastereomer of **12**: [α]^25^_D_ = +6.8 (*c* 0.53, CHCl_3_); ^1^H NMR (400 MHz, CDCl_3_) δ 7.15 (t, *J* = 7.9 Hz, 1H), 6.99 (d, *J* = 7.8 Hz, 1H), 6.94 (d, *J* = 6.7 Hz, 2H), 5.83–5.67 (m, 1H), 4.94 (dd, *J* = 3.4, 1.6 Hz, 1H), 4.90 (s, 1H), 3.96–3.80 (m, 2H), 2.82–2.76 (m, 1H), 2.70–2.58 (m, 1H), 2.31 (s, 3H), 1.95–1.74 (m, 3H), 1.74–1.60 (m, 1H), 1.21 (d, *J* = 6.9 Hz, 3H), 0.98 (t, *J* = 7.1 Hz, 3H); ^13^C NMR (100 MHz, ) δ 175.6, 142.4, 138.5, 137.5, 129.1, 128.0, 127.1, 125.2, 114.6, 60.0, 48.1, 46.1, 30.9, 21.5, 14.9, 13.9; HRMS (EI) *m*/*z* calcd for C_17_H_24_O_2_ (M^+^) 260.1776, found 260.1775.

**2.2.31 (3*S*,*E*)-Ethyl 8-bromo-2,7-dimethyl-3-(*m*-tolyl)oct-6-enoate (10)**

Compound **10** was synthesized from a diastereomeric mixture of **12** following the procedure described for compound **3a** in 70% yield and subjected to the next step without separation of its isomers. Characterization data of **10** was obtained by performing the CM of methallyl bromide with each of the two diastereomers of **12** separately. Data for **10** synthesized from the major isomer of **12**: ^1^H NMR (600 MHz, CDCl_3_) δ 7.20–7.16 (m, 1H), 7.03 (t, *J* = 6.3 Hz, 1H), 6.96–6.88 (m, 2H), [5.49 (t, *J* = 7.0 Hz), 5.29 (ddd, *J* = 7.5, 7.5, 1.5 Hz) 1H], 4.18 (q, *J* = 7.1 Hz, 2H), [3.92 (s), 3.78–3.72 (m), 2H], 2.75–2.66 (m, 1H), 2.63–2.55 (m, 1H), [2.34 (s), 2.33 (s), 3H], 1.85–1.72 (m, 2H), 1.72–1.60 (m, 2H), 1.54 (s, 3H), 1.29 (t, *J* = 7.1 Hz, 3H), 0.89 (d, *J* = 7.0 Hz, 3H); ^13^C NMR (150 MHz, CDCl_3_) δ 176.5, 141.6, 137.9, 132.2, 131.0, 129.2, 128.3, 127.4, 125.3, 60.3, 48.6, 46.1, 41.8, 33.8, 26.2, 21.5, 16.3, 14.5, 14.3; HRMS (EI) *m*/*z* calcd for C_19_H_27_BrO_2_ (M^+^) 366.1194, found 366.1191. Data for **10** synthesized from the minor isomer of **12**: ^1^H NMR (400 MHz, CDCl_3_) δ 7.22–7.09 (m, 1H), 7.03–6.80 (m, 3H), 5.53 (t, *J* = 6.4 Hz, 1H), 3.93 (s, 2H), 3.90–3.80 (m, 2H), 2.81–2.71 (m, 1H), 2.69–2.53 (m, 1H), 2.31 (s, 3H), 1.89–1.75 (m, 3H), 1.74–1.61 (m, 1H), 1.56 (s, 3H), 1.20 (d, *J* = 6.9 Hz, 4H), 0.99 (t, *J* = 7.1 Hz, 3H); ^13^C NMR (100 MHz, ) δ 175.5, 142.1, 137.5, 132.3, 131.0, 129.2, 128.0, 127.2, 125.2, 59.9, 48.1, 46.1, 41.8, 31.0, 26.0, 21.4, 14.8, 14.5, 13.9; HRMS (EI) *m*/*z* calcd for C_19_H_27_BrO_2_ (M^+^) 366.1194, found 366.1186.

**2.2.32 (1*S*,2*R*,5*R*)-Ethyl 1-methyl-2-(prop-1-en-2-yl)-5-(*m*-tolyl)cyclopentanecarboxylate 11**

To a solution of allylic bromide **10** (50.0 mg, 0.14 mmol) in THF (17 mL) was added LiHMDS (0.68 mL, 1.0 M solution in THF) at 0 °C. The mixture was stirred for 24 h at room temperature, cooled to 0 °C, and quenched with saturated aqueous NH_4_Cl solution. The mixture was concentrated at reduced pressure and the residue was dissolved in EtOAc. The mixture was washed with brine, dried over anhydrous MgSO_4_, and concentrated at reduced pressure. The resulting residue was purified by flash column chromatography on silica gel (hexanes/EtOAc, 20:1) to give a diastereomeric mixture of **11**, **11-*iso***, and **11-*cis*** (29.6 mg; 5.5:2.8:1, by ^1^H 600 MHz NMR) in 76% yield. The three diastereomers were separated by NP-preparative HPLC. Data for cylopentanecarboxylate **11**: [α]^25^_D_ = +45.8 (*c* 0.045, CHCl_3_); ^1^H NMR (600 MHz, CDCl_3_) δ 7.15 (t, *J* = 7.4 Hz, 1H), 7.00 (d, *J* = 7.5 Hz, 1H), 6.96 (s, 1H), 6.96 (d, *J* = 8.1 Hz, 1H), 4.83 (s, 1H), 4.79 (s, 1H), 4.17–4.03 (m, 2H), 3.96 (dd, *J* = 11.7, 6.7 Hz, 1H), 2.47 (t, *J* = 8.5 Hz, 1H), 2.32 (s, 3H), 2.11–2.06 (m, 1H), 2.04–1.95 (m, 1H), 1.95-1.90 (m, 2H), 1.77 (s, 4H), 1.26 (t, *J* = 7.1 Hz, 3H), 0.91 (s, 3H); ^13^C NMR (150 MHz, CDCl_3_) δ 176.4, 145.3, 141.1, 137.4, 129.4, 127.8, 127.0, 125.6, 111.9, 60.4, 59.5, 55.7, 51.8, 30.0, 29.5, 23.3, 22.9, 21.5, 14.2; HRMS (EI) *m*/*z* calcd for C_19_H_26_O_2_ (M^+^) 286.1933, found 286.1930. Data for **11-*iso***: [α]^25^_D_ = −24.5 (*c* 0.073, CHCl_3_); ^1^H NMR (600 MHz, CDCl_3_) δ 7.15 (t, *J* = 7.8 Hz, 1H), 7.01 (d, *J* = 7.5 Hz, 1H), 6.93 (br s, 2H), 4.87 (s, 1H), 4.77 (s, 1H), 4.15 (q, *J* = 7.1 Hz, 2H), 3.73 (t, *J* = 10.1 Hz, 1H), 3.27 (t, *J* = 9.9 Hz, 1H), 2.30 (s, 3H), 2.13–2.08 (m, 2H), 2.01–1.95 (m, 2H), 1.65 (s, 3H), 1.25 (t, *J* = 7.1 Hz, 3H), 0.72 (s, 3H); ^13^C NMR (100 MHz, CDCl_3_) δ 177.5, 144.4, 139.6, 137.4, 129.1, 127.8, 127.4, 125.3, 111.9, 60.5, 55.6, 54.9, 54.8, 26.4, 25.5, 23.3, 21.5, 14.2, 11.2; HRMS (EI) *m*/*z* calcd for C_19_H_26_O_2_ (M^+^) 286.1933, found 286.1933. Data for **11-*cis***: [α]^25^_D_ = +15.0 (*c* 0.010, CHCl_3_); ^1^H NMR (400 MHz, CDCl_3_) δ 7.14 (t, *J* = 7.3 Hz, 1H), 7.00 (s, 1H), 6.99 (d, *J* = 7.0 Hz, 1H), 4.86 (s, 1H), 4.77 (s, 1H), 3.59–3.46 (m, 1H), 3.37 (dd, *J* = 12.6, 5.9 Hz, 1H), 2.93 (dd, *J* = 11.4, 6.9 Hz, 1H), 2.31 (s, 3H), 2.11–1.92 (m, 3H), 1.79–1.76 (m, 1H), 1.62 (s, 3H), 1.17 (s, 3H), 0.84 (t, *J* = 7.1 Hz, 3H); ^13^C NMR (150 MHz, CDCl_3_) δ 176.0, 145.0, 141.1, 137.1, 129.2, 127.7, 127.3, 125.5, 111.3, 60.1, 59.1, 55.0, 52.8, 30.8, 29.1, 23.5, 21.40, 21.38, 13.6; HRMS (EI) *m*/*z* calcd for C_19_H_26_O_2_ (M^+^) 286.1933, found 286.1930.

# Copies of the ^1^H and ^13^C NMR spectra

**Figure S1**. ^1^H NMR spectrum (400 MHz) of compound **3a** in CDCl_3_.

**Figure S2**. ^13^C NMR spectrum (100 MHz) of compound **3a** in CDCl_3_.

**Figure S3**. ^1^H NMR spectrum (400 MHz) of compound **3b** in CDCl_3_.

**Figure S4**. ^13^C NMR spectrum (150 MHz) of compound **3b** in CDCl_3_.

**Figure S5**. ^1^H NMR spectrum (400 MHz) of compound **5a** in CDCl_3_.

**Figure S6**. ^13^C NMR spectrum (100 MHz) of compound **5a** in CDCl_3_.

**Figure S7**. ^1^H NMR spectrum (400 MHz) of compound **15a** in CDCl_3_.

**Figure S8**. ^13^C NMR spectrum (150 MHz) of compound **15a** in CDCl_3_.

**Figure S9**. ^1^H NMR spectrum (400 MHz) of compound **5b** in CDCl_3_.

**Figure S10**. ^13^C NMR spectrum (100 MHz) of compound **5b** in CDCl_3_.

**Figure S11**. ^1^H NMR spectrum (400 MHz) of compound **15b** in CDCl_3_.

**Figure S12**. ^13^C NMR spectrum (100 MHz) of compound **15b** in CDCl_3_.

**Figure S13**. ^1^H NMR spectrum (400 MHz) of compound **5c** in CDCl_3_.

**Figure S14**. ^13^C NMR spectrum (100 MHz) of compound **5c** in CDCl_3_.

**Figure S15**. ^1^H NMR spectrum (400 MHz) of compound **15c** in CDCl_3_.

**Figure S16**. ^13^C NMR spectrum (100 MHz) of compound **15c** in CDCl_3_.

**Figure S17**. ^1^H NMR spectrum (400 MHz) of compound **5d** in CDCl_3_.

**Figure S18**. ^13^C NMR spectrum (100 MHz) of compound **5d** in CDCl_3_.

**Figure S19**. ^1^H NMR spectrum (400 MHz) of compound **15d** in CDCl_3_.

**Figure S20**. ^13^C NMR spectrum (150 MHz) of compound **15d** in CDCl_3_.

**Figure S21**. ^1^H NMR spectrum (400 MHz) of compound **5e** in CDCl_3_.

**Figure S22**. ^13^C NMR spectrum (150 MHz) of compound **5e** in CDCl_3_.

**Figure S23**. ^1^H NMR spectrum (600 MHz) of compound **15e** in CDCl_3_.

**Figure S24**. ^13^C NMR spectrum (150 MHz) of compound **15e** in CDCl_3_.

**Figure S25**. ^1^H NMR spectrum (400 MHz) of compound **5f** in CDCl_3_.

**Figure S26**. ^13^C NMR spectrum (100 MHz) of compound **5f** in CDCl_3_.

**Figure S27**. ^1^H NMR spectrum (600 MHz) of compound **15f** in CDCl_3_.

**Figure S28**. ^13^C NMR spectrum (150 MHz) of compound **15f** in CDCl_3_.

**Figure S29**. ^1^H NMR spectrum (600 MHz) of compound **5g** in CDCl_3_.

**Figure S30.** ^13^C NMR spectrum (150 MHz) of compound **5g** in CDCl_3_.

**Figure S31**. ^1^H NMR spectrum (600 MHz) of compound **15g** in CDCl_3_.

**Figure S32.** ^13^C NMR spectrum (150 MHz) of compound **15g** in CDCl_3_.

**Figure S33**. ^1^H NMR spectrum (400 MHz) of compound **5h** in CDCl_3_.

**Figure S34**. ^13^C NMR spectrum (100 MHz) of compound **5h** in CDCl_3_.

**Figure S35**. ^1^H NMR spectrum (400 MHz) of compound **15h** in CDCl_3_.

**Figure S36**. ^13^C NMR spectrum (100 MHz) of compound **15h** in CDCl_3_.

**Figure S37**. ^1^H NMR spectrum (600 MHz) of compound **7a** in CDCl_3_.

**Figure S38**. ^13^C NMR spectrum (150 MHz) of compound **7a** in CDCl_3_.

**Figure S39**. ^1^H NMR spectrum (600 MHz) of compound **16a** in CDCl_3_.

**Figure S40**. ^13^C NMR spectrum (150 MHz) of compound **16a** in CDCl_3_.

**Figure S41**. ^1^H NMR spectrum (400 MHz) of compound **7b** in CDCl_3_.

**Figure S42**. ^13^C NMR spectrum (100 MHz) of compound **7b** in CDCl_3_.

**Figure S43**. ^1^H NMR spectrum (400 MHz) of compound **16b** in CDCl_3_.

**Figure S44**. ^13^C NMR spectrum (100 MHz) of compound **16b** in CDCl_3_.

**Figure S45**. ^1^H NMR spectrum (400 MHz) of compound **7c** in CDCl_3_.

**Figure S46**. ^13^C NMR spectrum (150 MHz) of compound **7c** in CDCl_3_.

**Figure S47**. ^1^H NMR spectrum (400 MHz) of compound **16c** in CDCl_3_.

**Figure S48**. ^13^C NMR spectrum (150 MHz) of compound **16c** in CDCl_3_.

**Figure S49**. ^1^H NMR spectrum (400 MHz) of compound **7d** in CDCl_3_.

**Figure S50**. ^13^C NMR spectrum (100 MHz) of compound **7d** in CDCl_3_.

**Figure S51**. ^1^H NMR spectrum (400 MHz) of compound **16d** in CDCl_3_.

**Figure S52**. ^13^C NMR spectrum (100 MHz) of compound **16d** in CDCl_3_.

**Figure S53**. ^1^H NMR spectrum (600 MHz) of compound **7e** in CDCl_3_.

**Figure S54**. ^13^C NMR spectrum (150 MHz) of compound **7e** in CDCl_3_.

**Figure S55**. ^1^H NMR spectrum (600 MHz) of compound **16e** in CDCl_3_.

**Figure S56**. ^13^C NMR spectrum (150 MHz) of compound **16e** in CDCl_3_.

**Figure S57**. ^1^H NMR spectrum (600 MHz) of compound **14** in CDCl_3_.

**Figure S58**. ^13^C NMR spectrum (150 MHz) of compound **14** in CDCl_3_.

**Figure S59**. ^1^H NMR spectrum (400 MHz) of compound **12** (the major isomer) in CDCl_3_.

**Figure S60**. ^13^C NMR spectrum (100 MHz) of compound **12** (the major isomer) in CDCl_3_.

**Figure S61**. ^1^H NMR spectrum (400 MHz) of compound **12** (the minor isomer) in CDCl_3_.

**Figure S62**. ^13^C NMR spectrum (100 MHz) of compound **12** (the minor isomer) in CDCl_3_.

**Figure S63**. ^1^H NMR spectrum (600 MHz) of compound **10** in CDCl_3_.

**Figure S64**. ^13^C NMR spectrum (150 MHz) of compound **10** in CDCl_3_.

**Figure S65**. ^1^H NMR spectrum (400 MHz) of compound **10** (minor isomer) in CDCl_3_.

**Figure S66**. ^13^C NMR spectrum (100 MHz) of compound **10** (minor isomer) in CDCl_3_.

**Figure S67**. ^1^H NMR spectrum (600 MHz) of compound **11** in CDCl_3_.

**Figure S68**. ^13^C NMR spectrum (150 MHz) of compound **11** in CDCl_3_.

**Figure S69**. ^1^H NMR spectrum (600 MHz) of compound **11-*iso*** in CDCl_3_.

**Figure S70**. ^13^C NMR spectrum (100 MHz) of compound **11-*iso*** in CDCl_3_.

**Figure S71**. ^1^H NMR spectrum (400 MHz) of compound **11-*cis*** in CDCl_3_.

**Figure S72**. ^13^C NMR spectrum (150 MHz) of compound **11- *cis*** in CDCl_3_.
